# Structural and Electronic Response of Multigap N-Doped In_2_Se_3_: A Prototypical Material for Broad Spectral Optical Devices

**DOI:** 10.1021/acsami.4c08610

**Published:** 2024-09-06

**Authors:** Guilherme Rodrigues-Fontenele, Gabriel Fontenele, Mirela R. Valentim, Luisa V. C. Freitas, Gilberto Rodrigues-Junior, Rogério Magalhães-Paniago, Angelo Malachias

**Affiliations:** † Physics Department, Federal University of Minas Gerais (UFMG), Belo Horizonte, Minas Gerais 30123-970, Brazil; ‡ Institute of Physics, State University of Campinas (UNICAMP), Campinas, São Paulo 13083-859, Brazil; § Physics Department, Federal University of Viçosa (UFV), Viçosa, Minas Gerais 36570-900, Brazil

**Keywords:** indium selenide, layered semiconductors, n-doping system, scanning tunneling spectroscopy, crystal truncation rod

## Abstract

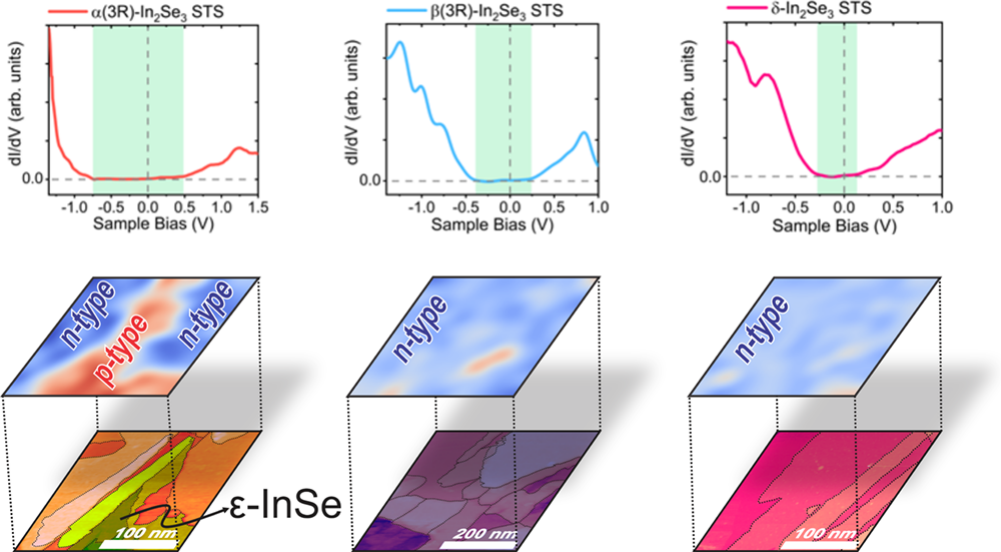

The production of controlled doping in two-dimensional semiconductor materials is a challenging issue when introducing these systems into current and future technology. In some compounds, the coexistence of distinct crystallographic phases for a fixed composition introduces an additional degree of complexity for synthesis, chemical stability, and potential applications. In this work, we demonstrate that a multiphase In_2_Se_3_ layered semiconductor system, synthesized with three distinct structures—rhombohedral α and β-In_2_Se_3_ and trigonal δ-In_2_Se_3_—exhibits chemical stability and well-behaved n-type doping. Scanning tunneling spectroscopy measurements reveal variations in the local electronic density of states among the In_2_Se_3_ structures, resulting in a compound system with electronic bandgaps that range from infrared to visible light. These characteristics make the layered In_2_Se_3_ system a promising candidate for multigap or broad spectral optical devices, such as detectors and solar cells. The ability to tune the electronic properties of In_2_Se_3_ through structural phase manipulation makes it ideal for integration into flexible electronics and the development of heterostructures with other materials.

## Introduction

The investigation of layered III–VI semiconductors continues to attract significant interest due to their potential applications in nano-optoelectronic devices, driven by their atomic-scale thickness, outstanding light absorption properties, high carrier mobility and layer-dependent bandgaps.^1−6^ Among III–VI semiconductors, In_2_Se_3_ is a promising material thanks to its structural diversity, typically exhibiting multiple layered polymorphs (α, β, δ, and κ) and other polytypes. These structural variations significantly influence the electronic properties of In_2_Se_3_, resulting in possible higher-order topological states, in-plane and out-of-plane ferroelectricity and a broad range of electronic bandgaps.^7−10^ As a result, In_2_Se_3_ is exceptionally well-suited for applications such as solar cells, high-performance photodetectors, nonvolatile memories, ferroelectric field-effect transistors (FeFETs), gas sensors, and more.^11−20^

In recent years, the exploration of α, β, and δ-In_2_Se_3_ has been focused on fundamental properties and device applications as an n-type material, usually employed as single-phase device prototypes. Typically, suitable semiconductor systems for applications are designed as single crystals with well-determined bandgap energy and well-known doping levels. Such prerogatives were partially softened in the past with the emergence of polycrystalline nitrides, which allowed the development of blue and white light-emitting diodes.^21−23^ In certain compounds, these disadvantages are coupled with a complex chemical and structural phase diagram, as seen in Bi-Se and Bi-Te materials, adding an undesired degree of freedom.^24−28^ With the recent intensification of research in layered (or two-dimensional) materials, several limiting factors were unveiled. For instance, intrinsic difficulties for controlled doping, the strong influence of substrate/support proximity and point defects (vacancies) on the final performance, and stability concerning processing methods required for device fabrication (lithography, electric contact, encapsulation).^29−37^

Our work presents properties of a multiphase In_2_Se_3_ sample, focusing on the potential of devices with phase coexistence, particularly using the δ-polymorph as a narrow-bandgap material. In this sense, it is crucial to revise the properties of single-phase devices. In the following paragraphs, we depict a brief revision of some of the most recent works, providing a concise scenario of the use of α and β-In_2_Se_3_ layered structures. No experimental reports of the electronic properties and device applications of δ-In_2_Se_3_ were retrieved in the literature, although electronic structure calculations were found.^38^

Zhang et al. observed an energy bandgap variation by bending an α-In_2_Se_3_ monolayer, showing that substrate defects can be used as a tuning strategy for device fabrication.^39^ Liu et al demonstrated that, by introducing hole-doping into an α-In_2_Se_3_ monolayer system, ferromagnetic properties are also induced.^40^ This work observed that a minor strain in hole-doped α-In_2_Se_3_ induces a ferromagnetic state, making it suitable for multifield sensor devices and forthcoming spintronic applications. Such ferroelectric capability of α-In_2_Se_3_ has been explored as an interesting phenomenon for applications that range from ferroelectric-modulated photodetectors to nonvolatile memory devices. Jia et al. developed an α-In_2_Se_3_/Si heterostructure photodetector, where the robust ferroelectric polarization of α-In_2_Se_3_ enhanced the modulation of the device.^41^ Zou et al. also investigated an α-In_2_Se_3_-based photodetector, with spectral response in the visible wavelength range, spanning from 405 to 905 nm, using WSe_2_ as the p-type material (providing an optimized underwater optical communication system that detects modulated light signals).^42^ Another α-In_2_Se_3_/WSe_2_ photodetector was investigated by Zhao et al., showing an enhancement of the photocurrent by 18 times under tensile strain application.^43^ Dutta et al. designed a ferroelectric semiconductor field-effect device based on α-In_2_Se_3_ and MoS_2_ heterostructure.^44^ Their findings suggest that n–i and n–i–n junctions are facilitated by in-plane and out-of-plane ferroelectric properties of α-In_2_Se_3_. Li et al. fabricated ferroelectric semiconductor field-effect transistors and few-layers graphene/α-In_2_Se_3_/few-layers graphene heterostructure.^45^ The device demonstrated high on/off performance and large storage capacity. Nahid et al. also fabricated a graphene/α-In_2_Se_3_/graphene device, focusing on investigating of the ferroelectric photovoltaic effect.^46^

The design of heterostructures based on the β-polymorph of In_2_Se_3_ has been also intensively investigated, with particular attention driven toward its high electrical conductivity and low thermal conductivity.^47^ Shao et al. fabricated a heterostructure composed of β-In_2_Se_3_ layers deposited onto a monolayer MoS_2_.^48^ Their findings revealed that the edge of this heterostructure exhibits exceptional electrocatalytic activity for hydrogen evolution reaction (HER). They also highlight that the introduction of β-In_2_Se_3_ potentially improves the water adsorption of their device, thereby improving HER performance. Wu et al. also highlighted the use of the β-In_2_Se_3_ monolayer as an anode in alkaline-ion batteries.^49^ Their study indicates that a monolayer β-structure enhances the absorption of alkali metal ions more effectively than other reported materials. In 2024, Xiong et al. proposed a polarization-sensitive infrared photodetector based on a β-In_2_Se_3_/Te heterostructure.^50^ They emphasize the potential of this device for ASCII code transmission and polarization-sensitive infrared imaging. Claro et al. successfully fabricated a photodetector using β-In_2_Se_3_ on a c-sapphire.^51^ They reported an infrared response time of 7 ms, an improvement compared to previous β-In_2_Se_3_ photodetectors. Wan et al. prototyped a nonvolatile ferroelectric memory with a β/α/β-In_2_Se_3_ heterostructure built laterally.^52^ Their device demonstrated exceptional long-term data retention, required to maintain data integrity over extended periods, while also showcasing enhanced endurance performance.

In this work, we study a compound layered (two-dimensional phases) semiconductor system with chemical stability and well-behaved doping. We particularly show experimental results of bandgap and doping of the δ-In_2_Se_3_ phase. The resulting multiphase α, β, and δ-In_2_Se_3_ system have presented the required surface stability, probed by scanning tunneling microscopy and spectroscopy (STM/STS), crystal truncation rods (CTR) scattering and electron backscatter diffraction (EBSD), potentially allowing the development of high-performance wide spectral-range devices. In other words, the inherent polytypism of In_2_Se_3_, often viewed as an issue in device fabrication, can be strategically harnessed. According to our measurements, these phases present complementary bandgap ranging from 0.34 to 1.25 eV, with a phase distribution that can be optimized to meet specific requirements of In_2_Se_3_-based devices.

### Compound Details

**Figure 1:**
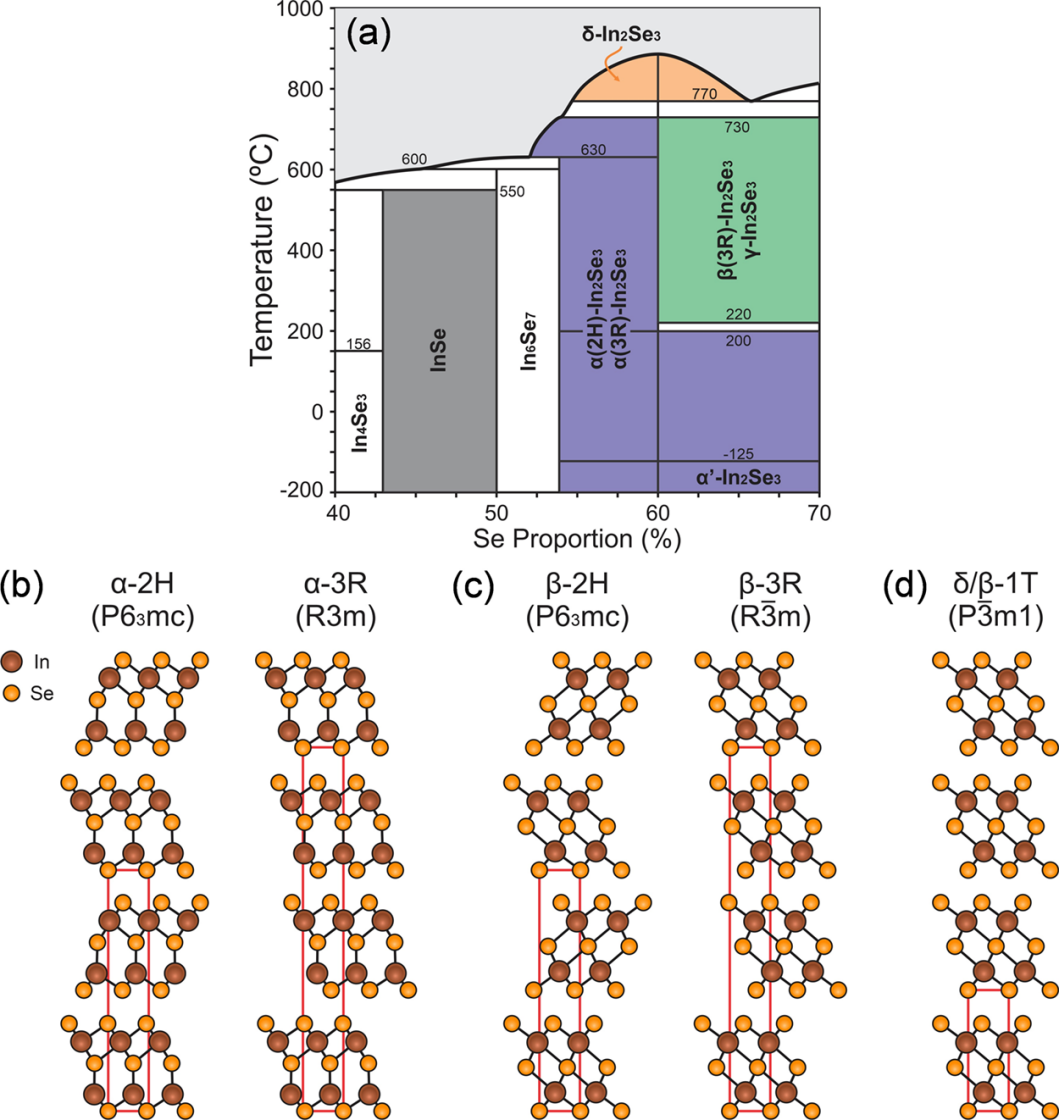
(a) Phase diagram of the In-Se system from 40 to 70% of Se atomic proportion. Figure adapted from ref (52). (b) Atomic structure representation along the *c*-direction of hexagonal and rhombohedral α-In_2_Se_3_ and (c) β-In_2_Se_3_ and (d) trigonal δ-In_2_Se_3_.

The growth of In_2_Se_3_ presents inherent complexity due to the rich phase diagram for In-Se compounds, adapted from ref (52), where the complete diagram is shown and reproduced in Figure 1a. One observes that the 2:3 stoichiometric proportion can be obtained along a considerably broad range of In-Se compositions, with some loci where more than one crystallographic phase can be synthesized. In this case, a synthesis that crosses the liquid–solid diagram frontier may pass through distinct phases along the quenching process. The nucleation of nonstoichiometric seeds along the cooling process can also induce the formation of neighboring stoichiometries with respect to the intended content. Since we are particularly interested in In_2_Se_3_, the structural and electronic properties of possible compounds with this In-Se ratio must be discussed.

Indium(III) selenide (In_2_Se_3_) exhibits at least three stable room-temperature layered structures, denoted as α-, β-, and δ-In_2_Se_3_. These layered polymorphs consist of monatomic layers of indium (In) and selenium (Se) arranged in a stacking sequence of Se-In-Se-In-Se, forming a quintuple-layer (QL) structure. Inside QLs, monatomic layers are held together through covalent bonds, while van der Waals forces mediate the interaction between stacked QLs.^53−56^ The distinct In_2_Se_3_ structures result from differences in stacking patterns of atoms within QLs, leading to slightly modified symmetries. For instance, α-In_2_Se_3_ exhibits at least two stacking variants: hexagonal (2H) and rhombohedral (3R) structures, characterized by space groups *P*63*mc* and *R*3*m*, respectively. β-In_2_Se_3_ exhibits hexagonal (2H) and rhombohedral (3R) variants, along with a trigonal (1T) one, with space groups *P*6_3_*mc*, *R*3̅*m*, and *P*3̅*m*1, respectively. In both α(3R)- and β(3R)-In_2_Se_3_, a translation along the *ab*-plane occurs in every alternate quintuple-layer, while the quintuple-layers of α(2Η)- and β(2Η)-In_2_Se_3_ rotate around the *c*-axis for every other QL. Lastly, the δ-In_2_Se_3_ is also referred to as β(1T) in other works, where the quintuple-layers remain unchanged along the c-direction.^38,57,58^ The crystalline structures of α-, β-, and δ-In_2_Se_3_ and their possible polytypes are illustrated in Figure 1b–d.

**Figure 2:**
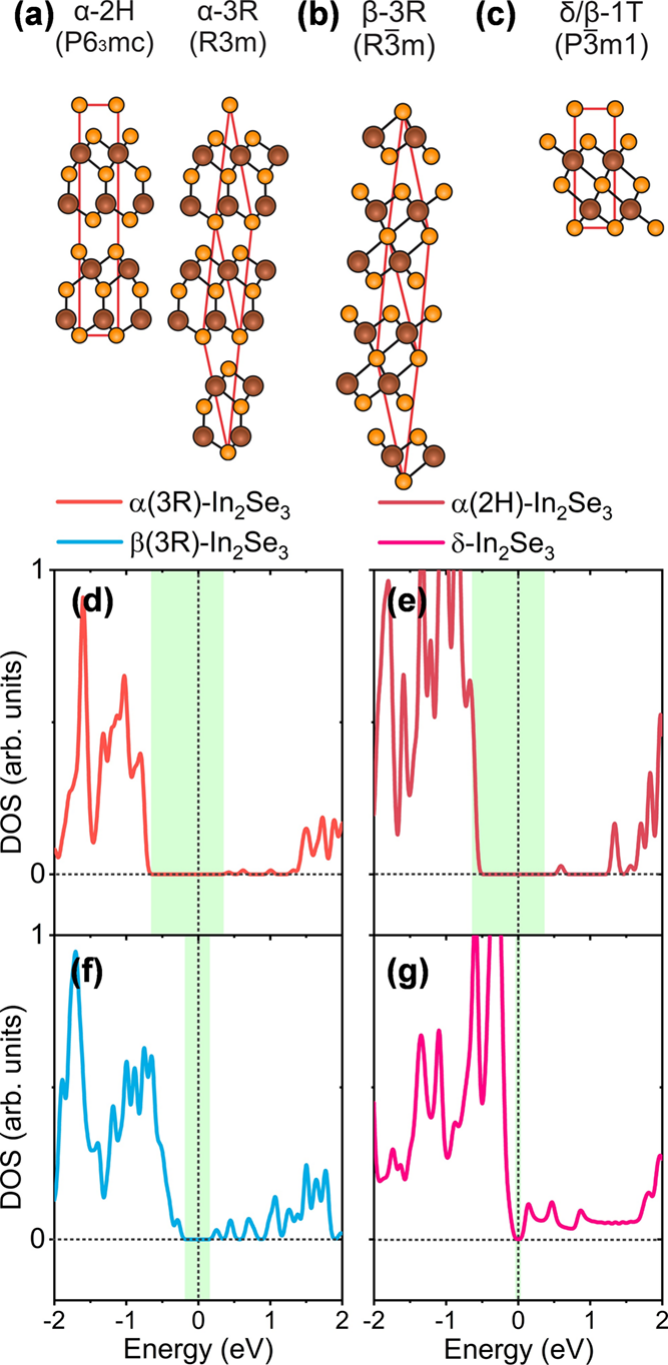
Primitive cell of In_2_Se_3_ structures calculated in this work. (a) α(2H) and α(3R)-In_2_Se_3_, (b) β(3R)-In_2_Se_3_ and (c) δ-In_2_Se_3_. Electronic density of states calculations of (d) α(3R)-In_2_Se_3_, (e) α(2H)-In_2_Se_3_, (f) β(3R)-In_2_Se_3_, and (g) δ-In_2_Se_3_.

Electronic band structure calculations using density functional theory (DFT) have been previously reported in the literature for α(3R), β(3R), and δ-In_2_Se_3_.^38,59^ For completeness, we have also carried out calculations using Heyd–Scuseria–Ernzerhof (HSE06) hybrid functional for the α(3R), α(2H), β(3R), and δ-In_2_Se_3_ phases. The corresponding electronic band structure calculations are provided in the Supporting Information (Figures S3 and S4), along with α(2H)-In_2_Se_3_ calculations (not reported in previous studies). We illustrate the primitive cell of all calculated In_2_Se_3_ structures in Figure 2a–c. Since scanning tunneling spectroscopy measurements provide the local density of states (LDOS) of studied materials, we present the density of states (DOS) for the abovementioned phases in Figure 2d–g for comparative analysis.

## Results

**Figure 3:**
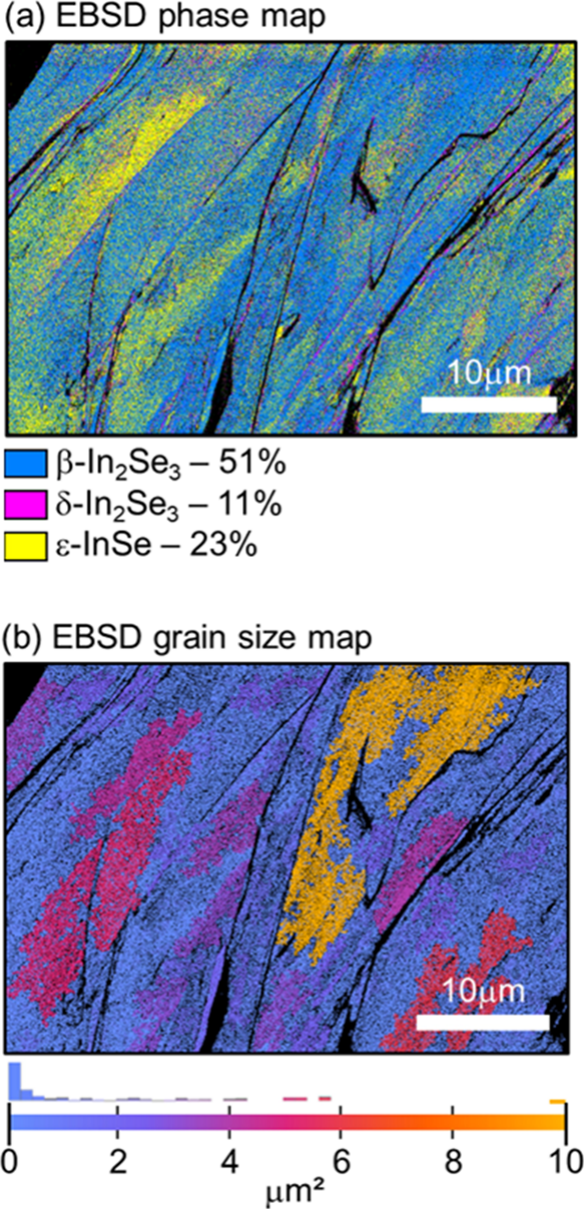
Electron backscatter diffraction measurements of our In-Se sample. (a) Phase map. This map shows a predominant region of β(3R)-In_2_Se_3_, with 51%, followed by 11% for δ-In_2_Se_3_, and 23% for ε-InSe. No α(3R)-In_2_Se_3_ was found in this region. (b) EBSD grain size map. The retrieved average grain size for this region is 1.72 μm^2^.

In a polymorphic sample, it is mandatory to understand the distribution of phases, as well as their volumetric content. Such information was experimentally retrieved here using electron backscatter diffraction (EBSD) and powder X-ray diffraction (PXPD). In Figure 3a, we show the EBSD phase map in a micrometer region of our sample. The result points out a coexistence of grains of different phases that varies from region to region due to the intrinsic local nature of the technique. We observe that the surface is predominantly composed of β(3R)-In_2_Se_3_, with a distribution proportion of 51% for this region. We only obtained 11% of δ-In_2_Se_3_ in this field of view, which is shown to be very distinct from the bulk proportion (see following paragraphs describing PXRD). Finally, the second most relevant phase obtained in Figure 3a is ε-InSe, comprising 23% of the sample in this region. Additional EBSD grain size analysis is provided in Figure 3b. As seen by the color scale, grain sizes within the analyzed area range from a few nanometers to a few micrometers, with an average value of 1.72 μm^2^.

**Figure 4:**
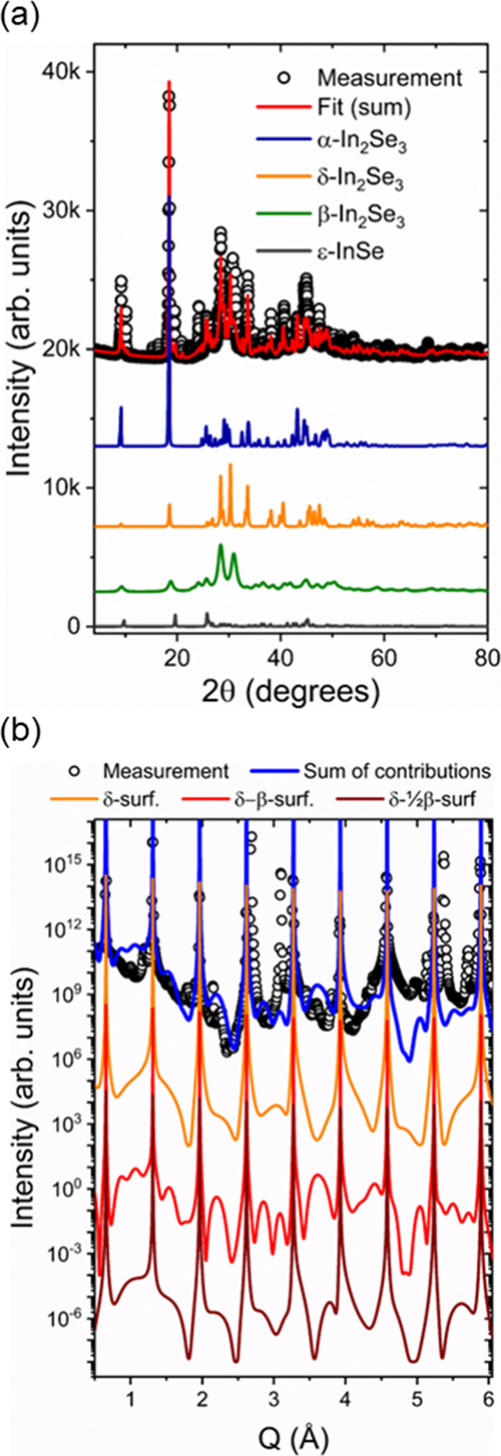
(a) Powder X-ray diffraction carried out in our sample. (b) Experimental CTR data along the (00*L*) direction depicted in black circles. A theoretical fit for β-In_2_Se_3_ (red), δ-In_2_Se_3_ (blue) and a composition of both phases (purple) are presented. The sum of contributions is shown in blue line.

Since EBSD can only be used to show local characteristics, one needs a statistically relevant technique. In Figure 4a, we show the powder X-ray diffraction pattern, obtained using Cu-Kα radiation in laboratory equipment, and the respective Rietveld refinement obtained for our sample. The refinement method was used to fit the experimental data to possible polymorphs and polytypes of In_x_Se_y_, including pure indium and selenium. We could not detect pure In and Se diffraction peaks, as well as no other stoichiometry besides In_2_Se_3_ and a minor fraction of InSe. Also, we could not detect any percentage of hexagonal α and β structures. We determined that the bulk material consists of α(3R), β(3R) and δ-In_2_Se_3_, along with a minor fraction of ε-InSe. The β(3R)-In_2_Se_3_ structure is predominant in bulk material, constituting 47% of the sample. In addition, α(3R)- and δ-In_2_Se_3_ showed similar proportions, accounting for 27 and 20%, respectively. The ε-InSe comprises 6% of the bulk material. The crystallographic data are available in Supporting Information (Table S2). Finally, we obtained an average grain size of 3920, 102, and 755 nm for α(3R), β(3R), and δ-In_2_Se_3_ structures, respectively, while a value of 980 nm was retrieved for ε-InSe. Notice that the local character of EBSD measurements becomes clear in this analysis since α-In_2_Se_3_ was not retrieved in the explored regions where electron diffraction was mapped.

The crystallographic behavior of the surface, along with its composition concerning the most relevant terminations in our cleaved sample, were obtained using synchrotron crystal truncation rod (CTR) measurements along the (00*L*) direction. In this case, we looked for possible surface stabilization for both In_2_Se_3_ and InSe structures found in our PXRD experiment. We simulated the CTR pattern for these materials using the formalism introduced by Ian Robison.^60^ The sample was modeled as bulk In_2_Se_3_ with surface layers, which can be β(3R)-In_2_Se_3_ and δ-In_2_Se_3_ QLs. Despite sharing the same QLs, differing only in their stacking patterns, these two structures exhibit distinct electronic densities of states, giving rise to diffuse diffraction signal asymmetries in the measured CTR range. Combinations of these surface layers were modeled separately, with their atomic stack described as a list of atoms and coordinates, interfering with the diffraction intensity due to modified scattering amplitudes. The CTR measurement and the respective simulations are shown in Figure 4b. Some of the peaks of the experimental data set that were not retrieved in the CTR simulation may correspond to β(2H)-In_2_Se_3_. The (00*L*) CTR cannot be used to define the crystallographic conformation of this phase, therefore it was not added due to the lack of reference data in crystallographic databases.

Surface layers in our model are modified with distinct stackings of In_2_Se_3_ and InSe. In particular, the CTR data was fitted with a combination of four distinct surface terminations: (i) δ-In_2_Se_3_ double quintuple-layers at the surface; (ii) β(3R)-In_2_Se_3_ double quintuple-layers at the surface; (iii) δ- QL and β(3R)-In_2_Se_3_ QL at the surface and; (iv) δ-In_2_Se_3_ QL followed by two β(3R)-In_2_Se_3_ QLs at the surface. The list provided above is ordered as a function of the layer stack relevance to the CTR fit. To obtain the fit depicted by the orange curve of Figure 4b (the closest to the characteristics of our measurement profile), we combined the following surface fractions: (i) 40%, (ii) 10%, (iii) 30%, and (iv) 20% of the surface, respectively. Differences concerning PXRD may be due to the reduced beam size in CTR measurements (50 × 30 μm^2^ on average, since it changes along the beam direction as the sample angle is varied). Although this leads us to a similar condition as the one obtained by EBSD, CTR measurements reveal that along the layer stacking phase coexistence can take place, since stacking models with mild variations in the surface atomic arrangement are mandatory to fit the measured data. Therefore, in addition to the lateral distribution of phases in the submicrometer range, the stacking changes in the nanometer-range increase the complexity of phase distribution in this system.

**Figure 5:**
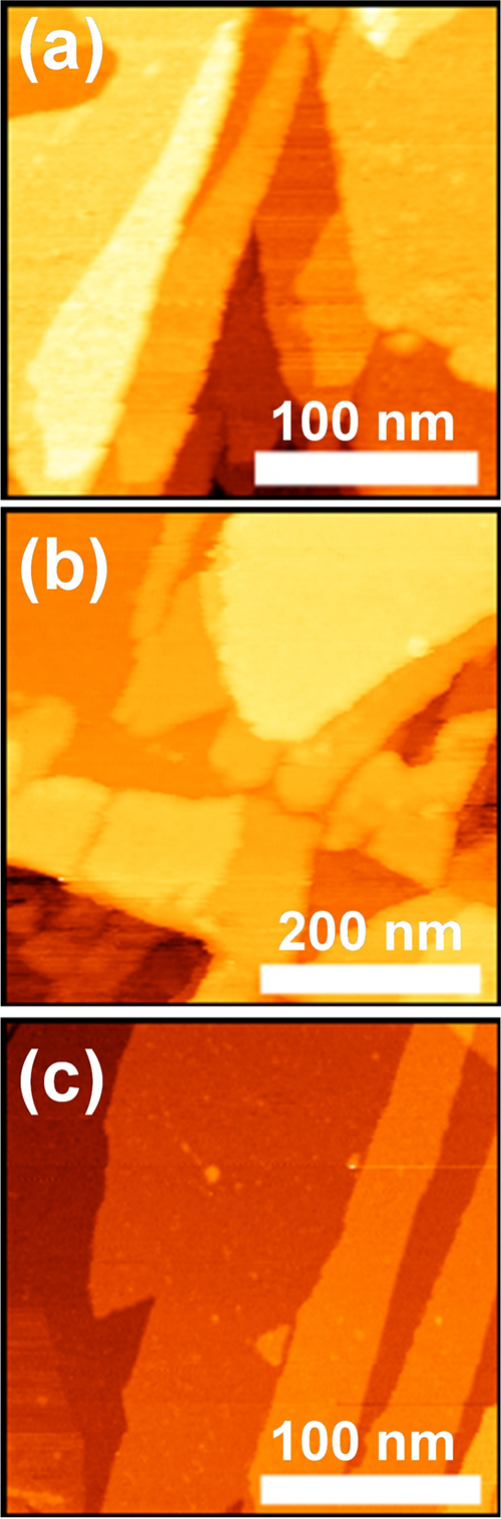
Scanning tunneling microscopy images at four distinct regions of our sample. The STM images were obtained using a sample bias of 1 V and current setpoints of 1 nA. Scan image areas are (a) 250 × 250 nm^2^ (b) 500 × 500 nm^2^, and (c) 250 × 250 nm^2^.

As In_2_Se_3_ polymorphs and polytypes can exhibit little structural differences and the grain sizes of our sample are usually of a fraction of microns, the combination of scanning tunneling microscopy and spectroscopy (STM/STS) can provide local topographic and electronic information sensitive to each investigated In_2_Se_3_ structure. We performed all STM and STS measurements using the same tip in an ultrahigh vacuum Omicron VT-STM microscope (see Methods). As expected from CTR and EBSD data, probing nanometer-scale areas using STS can facilitate the individual In_2_Se_3_ electronic band structure analysis, distinguishing the phase-dependent local electronic density of states. STM topographic images are shown in Figure 5a–c. By analyzing the height for all these steps (and consequently lamellae stacking), we retrieved heights that are multiples of approximately 1.00 nm for all In_2_Se_3_ polymorphs and 0.61 nm for ε-InSe. The theoretical value for QLs terminations is 0.971 nm for α polymorph and 0.978 for β/δ-In_2_Se_3_. Steps corresponding to ε-InSe terminations (0.61 nm) were also retrieved in a few STM and atomic force microscopy (AFM) measurements (see STM and AFM height profiles in the Supporting Information, Figures S7 and S8). However, this purely topographic analysis cannot unambiguously identify the observed In_2_Se_3_ polymorphs and their terminations, as step height closely aligns with all three In_2_Se_3_ quintuple-layers values.

From the previous height analysis, we noticed that in Figure 5a, there is an ambiguity when considering only the step height values (as seen in Figures S7 and S8). It is not possible, based solely on the topographical information, to determine if the terminations compatible with In_2_Se_3_/ε-InSe are ε-InSe or In_2_Se_3_. To resolve this, we performed a tunneling spectroscopic analysis using d*I*/d*V* curves. We observed the tunneling response of α(3R)-In_2_Se_3_ in some regions of Figure 5a, along with some terminations of ε-InSe.

**Figure 6:**
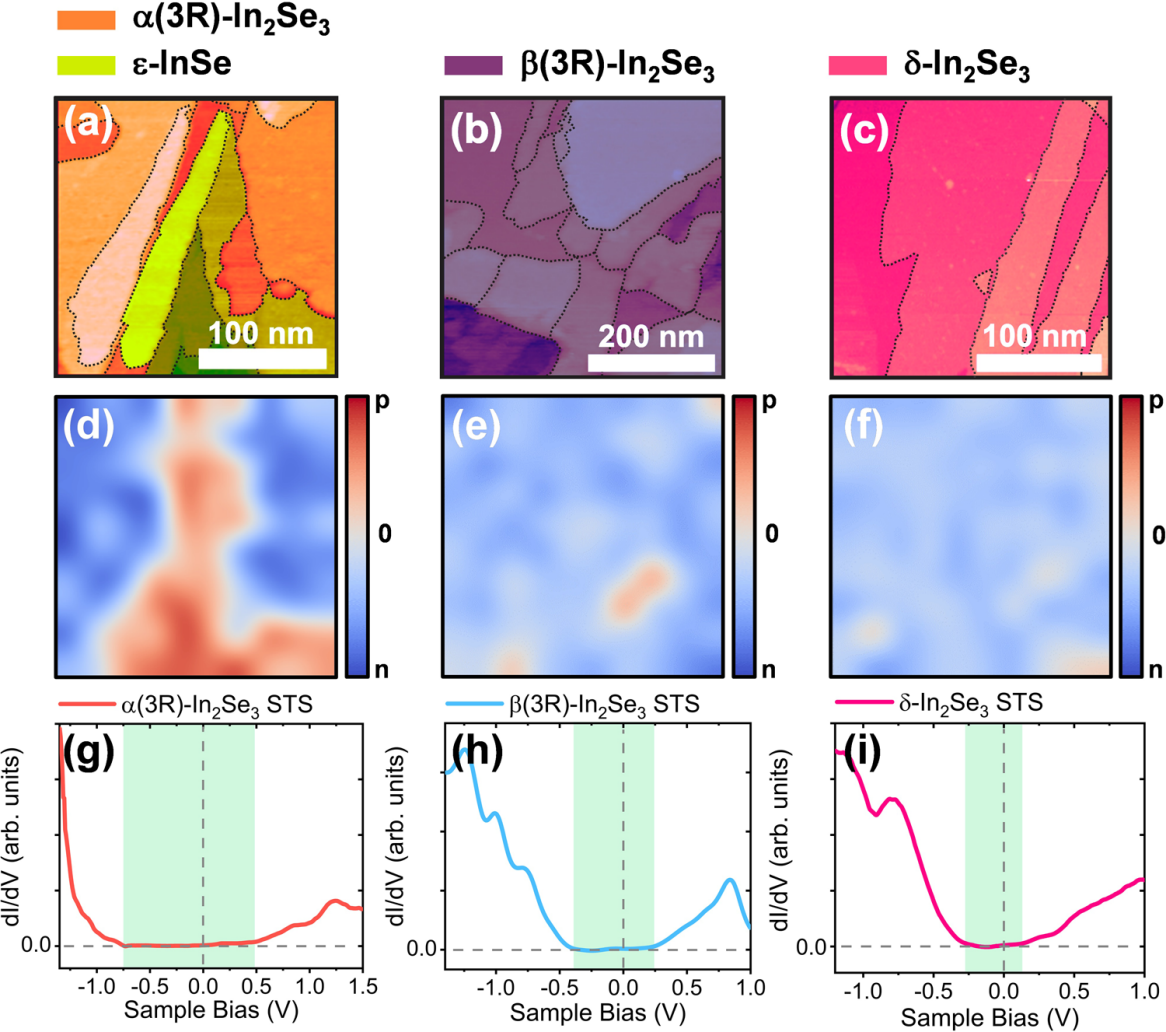
(a) Color-coded STM images displaying distinct In_2_Se_3_/InSe phases, with red shades indicating α(3R)-In_2_Se_3_ and green shades representing ε-InSe. (b) STM image with a pure β(3R)-In_2_Se_3_ domain colored in blue. (c) STM image with a pure δ-In_2_Se_3_ domain colored in pink. (d) Intrinsic doping map corresponding to the STS measurements in the same area of Figure 6a. One observes that the green area (ε-InSe) of the (a) panel exhibits p-type doping while the red area of panel (a) (α(3R)-In_2_Se_3_) exhibit n-type doping. (e) Doping map of Figure 6b showing n-type doping dominant behavior for the β(3R)-In_2_Se_3_ region of panel (b). (f) Doping map of the region of panel (c), a pure δ-In_2_Se_3_ domain, exhibiting n-type doping behavior. Selected scanning tunneling spectra are shown in the lower panels for (g) α(3R)-In_2_Se_3_, (h) β(3R)-In_2_Se_3_, and (i) δ-In_2_Se_3_.

In Figure 6 we provide color-coded STM images at the same regions of STM maps of Figure 5. Shades (tones) of colors were used in superposition with the original images to indicate the spatial location of each phase based on STS results, with step boundaries marked by black dashed contours. In Figure 6a green shades are used to identify ε-InSe regions, while red shades stand for α(3R)-In_2_Se_3_. In the images of Figure 6b,c, we observed domains of pure β(3R) (purple shades) and δ-In_2_Se_3_ (pink shades) phases, respectively, identified from STS measurements.

To investigate the intrinsic doping nature of In_2_Se_3_ polymorphs and ε-InSe, we generated an intrinsic doping map using tunneling spectroscopy data. By analyzing the Fermi level shift in our d*I*/d*V* curves, we observed a predominance of n-type doping in regions of α(3R)-In_2_Se_3_ and p-type doping in regions of ε-InSe, as shown in Figure 6d. The β(3R) and δ-In_2_Se_3_ domains in Figure 6b,c, respectively, also display well-defined n-type doping. In Figure 6e, the doping map corresponding to Figure 6b shows minimal doping variation, with a small p-type region near a grain boundary observed in the STM image. For δ-In_2_Se_3_, we also observe a well-defined intrinsic doping map with dominant n-type characteristics.

Selected characteristic tunneling spectra of α(3R), β(3R), and δ-In_2_Se_3_ are presented in Figure 6g–i. We extracted an average tunneling spectrum curve through STS grid analysis from previous STM images. In Figure 6g, one observes that the α(3R)-In_2_Se_3_ material has an electronic bandgap of approximately 1.25 eV. Under negative sample bias, a sharp peak rises toward quick saturation, indicating a large number of available electronic states. Positive bias leads to an ascending response with a mild slope. The retrieved bandgap matches our calculated value for α(3R)-In_2_Se_3_, differing by only 0.05 eV. We observe a qualitative agreement between the calculated electronic density of states (Figure 2d) for α(3R)-In_2_Se_3_ and the measured tunneling spectrum. In Figure 6h, we show the STS data for β(3R)-In_2_Se_3_, exhibiting a bandgap of 0.54 eV (DFT calculated bandgap for β(3R)-In_2_Se_3_ is 0.52 eV). Finally, Figure 6i shows the averaged STS data for δ-In_2_Se_3_. Despite differences from the α-polymorph, we observe similarities with the β(3R)-In_2_Se_3_ spectrum. The bandgap value obtained for this spectrum is reduced, matching the value calculated for δ-In_2_Se_3_ (0.34 eV for STS and 0.32 eV for DFT calculated DOS).

## Discussion

Once we realize that In_2_Se_3_ polymorphs and polytypes can be distinguished by their electronic response, one can proceed to cross-correlate measurements of different techniques. The most relevant information for a general overview of electronic properties in our system is the information about bandgap (*E*_G_) and intrinsic doping. Since STM/STS are local techniques, we have generated histograms of *E*_G_ and doping directly from d*I*/d*V* curves acquired in more than 3000 d*I*/d*V* curves (considering grid scans, line scans and point measurements), obtained in distinct regions of our sample. The extraction of *E*_G_ is carried out by establishing a threshold for zero d*I*/d*V* values (usually 2% of the maximum tunneling current range) and seeking the end points where the STS curve has nonzero values. For the doping level, one can consider the center of this *E*_G_ interval and its shift with respect to *V* = 0. In both cases, a histogram of *E*_G_ and intrinsic doping values is generated.

Despite our extensive set of measurements, it is conceivable that the scanned sample fraction may not accurately represent the proportions of each In_2_Se_3_ structure in our sample. To address this issue, we have normalized our histograms using the PXRD phase fractions, as PXRD covers a significant portion of the sample volume. The normalized results are discussed in the following paragraphs, while the raw histograms are presented in the Supporting Information (Figure S6). It is important to emphasize that the fits revealing bandgap (*E*_G_) peaks remain consistent in both raw and normalized data and do not impact our primary conclusions.

**Figure 7:**
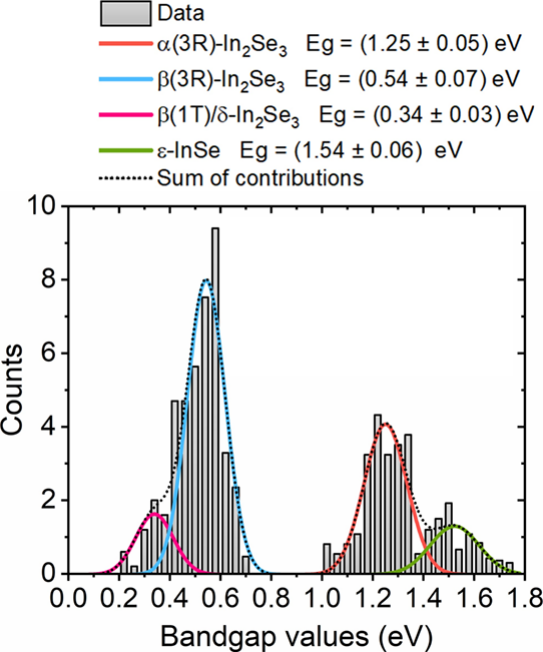
Energy bandgap values were obtained by analyzing the tunneling spectra. We observe a complementary bandgap for In_2_Se_3_ stoichiometry, ranging from 0.34 eV for δ-In_2_Se_3_ to 1.25 eV for α-In_2_Se_3_.

In Figure 7, we present the histogram of bandgap values obtained from the analysis of STS curves. It is possible to observe that the histogram does not correspond to simple monomodal or bimodal distributions, exhibiting complex behavior. In the interval comprising 0.2 eV < *E*_G_ < 0.8 eV one observes a strong peak near 0.6 eV with a well-defined shoulder approximately at 0.35 eV. In the *E*_G_ range that span from 1.0 to 1.8 eV a more defined bimodal distribution is retrieved, with peaks near 1.2 and 1.5 eV. Such peak profile allows a selection of energy gap values based on their occurrence in the synthesized material. It is possible then to group curves from distinct phases of In_2_Se_3_ and InSe and ensure statistical significance. The four observed prominent peaks and shoulder were then fitted with Gaussian functions. For the region of the histogram ranging from 0.2 to 0.7 eV, we retrieved Gaussian centers at *E*_G_ = 0.34 eV and *E*_G_ = 0.54 eV, corresponding to δ-In_2_Se_3_ and β(3R) phases, respectively. This energy range corresponds to the mid- and near-infrared spectrum, indicating a potential for photon absorption in this range. Subsequently, there are no counts for bandgap values between 0.7 and 1.0 eV. From 1.0 to 2.0 eV, we observed bandgap values corresponding to α(3R)-In_2_Se_3_, with the Gaussian peak centered at 1.25 eV and ε-InSe, with a Gaussian function centered at 1.54 eV (see ε-InSe electronic band structure calculations and STS analysis in Supporting Information, Figure S4).

**Figure 8:**
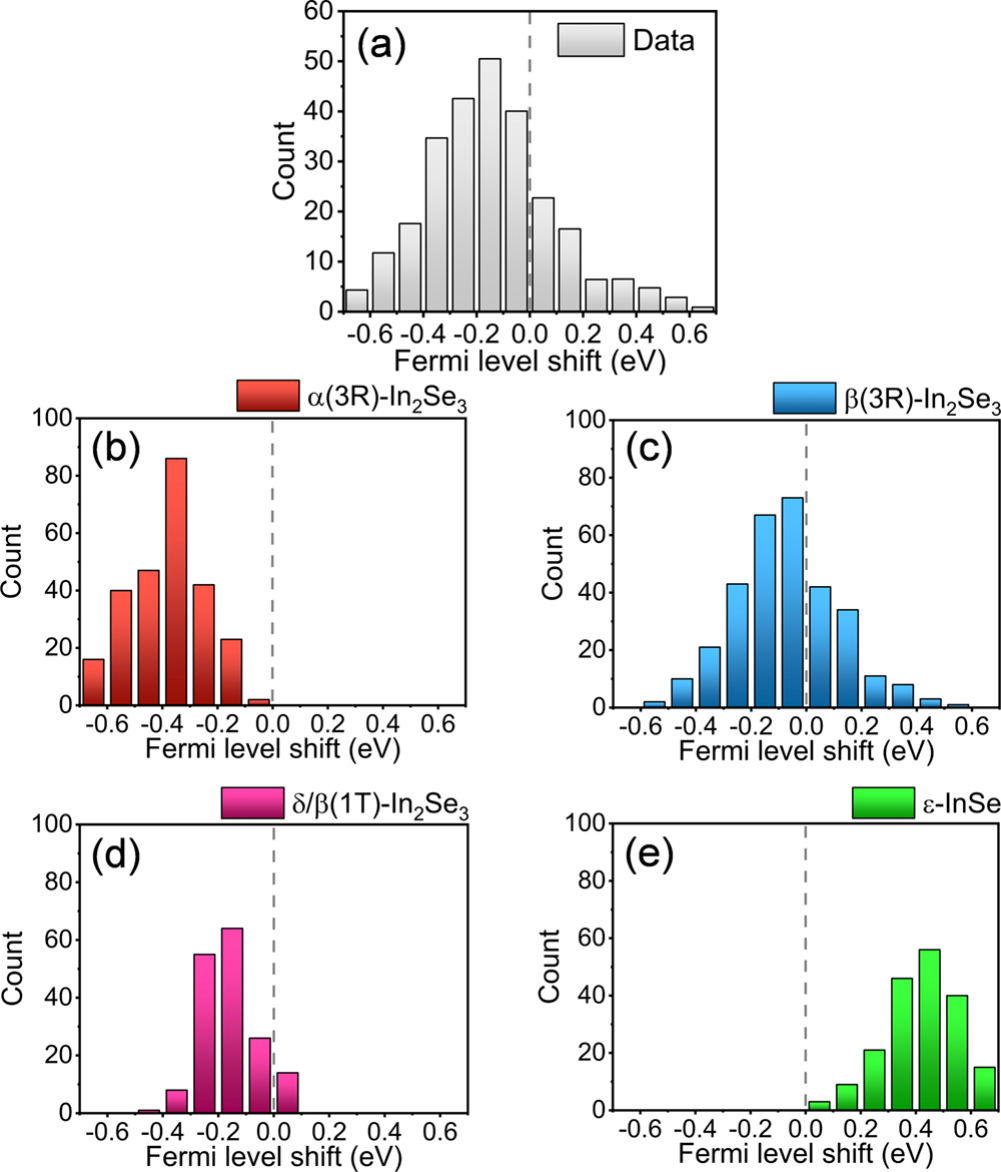
Intrinsic doping histogram evaluated by analyzing the Fermi level shift in STS measurements. (a) The sum of contributions of all structures in our sample: α(3R), β(3R), and δ-In_2_Se_3_, along with e-InSe. We also provide intrinsic doping analysis for each separated structure: (b) α(3R)-In_2_Se_3_, (c) β(3R)-In_2_Se_3_, (d) δ-In_2_Se_3_, and (e) ε-InSe. Apart from the ε-InSe phase, the In-Se with 2:3 stoichiometry presents an n-type behavior.

Once we have distinguished the phases using STS curve profiles and *E*_G_ values, we can proceed to evaluate the doping level of each phase separately. This procedure generates the histograms shown in Figure 8a–e. In panel (a), one can observe the overall doping distribution using the entire data set without any phase filtering. It becomes clear that most of the surface is n-doped, indicated by the shift of the histogram center toward negative bias values. However, we cannot establish if individual phases follow the same trend. By separating the information for each phase, we retrieve the histograms shown in panels (b–e). In all In_2_Se_3_ structures, n-type doping is observed, particularly pronounced in the α and δ structures, and less pronounced in the β(3R)-In_2_Se_3_. On the other hand, the ε-InSe (which represents a minor 6% fraction in the PXRD results) exhibits clear p-type doping behavior, as evidenced by the histogram center shifted toward positive bias values.

Such discriminated results strongly suggest that polymorphic In_2_Se_3_ can be used as n-doped layers in broad-spectrum infrared photodetectors and infrared-visible light solar cells. This is feasible since the bandgaps of probed phases cover a spectral range that starts at 0.4 eV and can absorb photons up to the visible light energies. For the InSe phase, it can be further minimized (or even suppressed) in future synthesis, providing a purely n-doped material and improving device performance.

## Conclusions

In conclusion, this work discusses the electronic and structural consequences of synthesizing a polymorphic In-Se system with 2:3 stoichiometry. The resulting material is potentially suitable for next-generation semiconductor-based devices due to its unique broad range of energy bandgaps, along with a phase-independent n-type intrinsic doping. By combining structural techniques – XPD, EBSD and CTR analysis – we were able to determine the volumetric and surface phase proportions for the existing compounds, revealing a coexistence of α(3R), β(3R), and δ-In_2_Se_3_, along with a minority of ε-InSe. This indicates that, besides the polytypic character of our samples, further refinement can be employed in order to suppress minor structural stacking variations, producing a crystal where the remaining phases are particularly advantageous for specific applications.

Our findings regarding the electronic response in our In_2_Se_3_ system revealed distinct bandgaps, ranging from 0.34 eV for δ-In_2_Se_3_ to 1.25 eV for α(3R)-In_2_Se_3_. The 2:3 stoichiometry is found as 94% of the synthesized volume (PXPD result) with intrinsic n-type doping retrieved by STS. These findings evidence the potential versatility of these materials in next-generation semiconductor devices with broad range infrared and near-infrared absorption. Thin layers with these compounds (obtained by, e.g., sputtering) can be a valuable addition to high-efficiency tandem solar cells.

An overview of our results and their relevance concerning previous experimental works is depicted in the following lines. First, we have shown that δ-In_2_Se_3_ is a complementary narrow-gap phase that keeps the usual n-doping of α and β-In_2_Se_3_ phases, providing a broader range of photonic interaction to future device development. This means that multiphase In_2_Se_3_ may be used as a versatile material, regardless of additional tunning variables such as external strain, subsequent doping procedures or substrate choice (that may be addressed for few-layer conditions in future works in the field). Phase stability at the surface was also found in our samples, since no structural or chemical degradation was detected by STM/STS, SEM, EBSD, or X-ray diffraction. Finally, if future procedures are established to control the occurrence of p-type doped ε-InSe phases, shown here but poorly explored in the literature, naturally self-assembled junctions can be made (or fully suppressed, depending on the desired applications).

One must mention that, as other two-dimensional materials, In_2_Se_3_ exhibit a layer-dependent bandgap. As the material thickness decreases to the few-layer or monolayer regime, electronic properties such as the bandgap, are significantly modified.^6,61,62^ This energy bandgap tunability adds another degree of freedom to the In_2_Se_3_ system. Such thickness-deterministic bandgap engineering, which can be tailored according to specific device requirements, is a remarkable advantage of In-Se compounds. Other electronic properties have been probed as the thickness of In_2_Se_3_ materials decreases, including ferroelectricity, piezoelectricity and higher-order topological states and must be explored for the δ-phase highlighted here.

### Methods

#### Crystal Growth

The In_2_Se_3_ crystal was grown using an evacuated and sealed quartz tube containing stoichiometric amounts of indium and selenium to crystallize the In_2_Se_3_ phase. The pressure inside the quartz tube during the sealing process was maintained at 70 milliTorr. After sealing the quartz tube with a hydrogen flame, the sealed tube was heated to 900 °C for a period of three days. Finally, the sample was allowed to cool down naturally to room temperature.

#### Structural Characterization

Scanning electron microscopy (SEM) images were acquired using a Hitachi TM4000Plus microscope. The microscope was operated at an accelerating voltage of 15 kV, using a secondary electron (SE) detector. The microscope is equipped with an energy-dispersive X-ray spectroscopy (EDS) detector by Oxford Instruments, facilitating compositional analysis of our sample. EDS measurements were conducted to determine the local atomic proportion in different regions within the sample, with the SEM image magnification consistently exceeding ×1.5k during the acquisition of the spectra. The set of curves was grouped and averaged to obtain a statistically meaningful representation.

X-ray Photoelectron Spectroscopy (XPS) was employed as a fundamental technique to analyze not only the chemical composition of the sample but also the oxidation states of indium and selenium. We used a SPECS PHOIBOS 100 hemispherical energy analyzer with an MCD Detector. All measurements presented in this work were performed in a vacuum environment (base pressure of 5 × 10^–10^ mbar) at room temperature. We utilized X-ray Kα photons emitted from an aluminum tube (1.486 keV) with a spot size of 0.2 × 0.2 mm^2^.

For our powder X-ray diffraction measurements, a Panalytical-Empyrean II diffractometer using Cu Kα_1_ radiation (λ = 0.1540 nm) at 45 kV and 40 mA was used. The experimental data underwent refinement using the Rietveld method. The refinement of the experimental data was analyzed for distinct In_x_Se_y_ crystallographic data structures, including InSe, In_2_Se_3_, In_4_Se_3_, pure In, and Se.

Electron backscatter diffraction measurements were conducted at the Laboratory of Microscopic Samples (LAM) of the Brazilian Synchrotron Light Laboratory (LNLS). A HELIOS 5 PFIB CXE DUALBEAM electron microscope equipped with an EBSD detector and dedicated analysis software was utilized. The measurements were conducted with a voltage of 18 kV, with the sample tilted at an angle of 70°.

Crystal truncation rod measurements were also conducted at LNLS, at the EMA (Extreme condition Methods of Analysis) beamline, using a 6-circle diffractometer. The X-ray wavelength used throughout the experiments was λ = 0.12915 nm and the beam size was 50 × 20 μm^2^.

#### Electronic Analysis

Scanning tunneling microscopy/spectroscopy (STM/STS) measurements were performed using an Omicron VT-STM microscope with an electrochemically etched tungsten tip. Additional electron beam etching of the tip was performed at 1000 V before measurements. STM and STS measurements were conducted at room temperature, with a base pressure of 2 × 10^–10^ mbar throughout the entirety of data acquisition. Tunneling spectra were acquired by scanning across various STM images, using always the same tip. To enhance the robustness of the analysis, an average of multiple curves from different points within the images was calculated.

Angle-Resolved PhotoEmission Spectroscopy (ARPES) measurements were performed at the SAPÊ beamline of the Brazilian Synchrotron Light Laboratory (LNLS). We used a SPECS PHOIBOS 150 spectrometer, a CARVING manipulator and a cryostat. The UV light source is a helium lamp with a beam size of 0.5 × 0.5 mm^2^ and photons in the energy range of 21 eV. The system was cooled using liquid nitrogen to the temperature of 77 K.

Finally, the electronic band structure and electronic density of state calculations presented in this work were carried out within the density functional theory (DFT) framework. We used an implementation of the screened hybrid functional of Heyd–Scuseria–Ernzerhof (HSE06) on the VASP package and projector augmented wave (PAW) technique to describe the interactions between valence electrons and ions.^63,64^ Structural optimization was performed until forces on the atoms were below the threshold of 0.3 eV/nm using a cutoff of 220 eV for the plane wave basis set.

## Supporting Information Available

Additional structural characterization of In_2_Se_3_ structures and ε-InSe using scanning electron microscopy, energy-dispersive X-ray spectroscopy, X-ray photoelectron spectroscopy and X-ray diffraction; electronic band structure calculations of α(3R), α(2H), β(3R), δ-In_2_Se_3_, and e-InSe; ARPES measurements; and surface termination analysis using STM height profiles (PDF)
