# Supplementary material for: Structural and Electronic Response of Multigap N-Doped In2Se3: A Prototypical Material for Broad Spectral Optical Devices
Source: ACS Appl Mater Interfaces. 2024 Sep 6;16(37):49902–12. doi: 10.1021/acsami.4c08610 (PMC11420874; doi:10.1021/acsami.4c08610)
Supplement: Supplementary file 1 — am4c08610_si_001.pdf [file am4c08610_si_001.pdf]

Supporting Information of

Structural and electronic response of multi-gap n-doped In_2Se_3: a prototypical material for broad spectral optical devices

Guilherme Rodrigues-Fontenele[1, ‡], Gabriel Fontenele[1, ‡], Mirela R. Valentim[2], Luisa V.C. Freitas[1], Gilberto Rodrigues-Junior[3], Rogério Magalhães-Paniago[1], Angelo Malachias[1, *]

1 Physics Department, Federal University of Minas Gerais (UFMG), Belo Horizonte, Minas Gerais, 30123-970, Brazil.

2 Institute of Physics, State University of Campinas (UNICAMP), Campinas, São Paulo, 13083-859, Brazil.

3 Physics Department, Federal University of Viçosa (UFV), Viçosa, Minas Gerais, 36570-900, Brazil.

‡ These authors contributed equally.

* Corresponding author: angelomalachias@gmail.com

Contents

***1.0 Structural and Chemical Characterization* 2**

1.1 Scanning Electron Microscopy and Energy-Dispersive X-ray Spectroscopy 2

1.2 X-ray Photoelectron Spectroscopy Analysis 2

1.3 X-ray diffraction 4

***2.0 Electronic Characterization* 5**

2.1 Density Functional Theory Calculations 5

2.2 Angle-Resolved Photoemission Spectroscopy Analysis 7

2.2 Scanning Tunneling Microscopy/Spectroscopy 8

1.0 Structural and Chemical Characterization

1.1 Scanning Electron Microscopy and Energy-Dispersive X-ray Spectroscopy

Indium selenide materials can crystallize in diverse stoichiometries (e.g. InSe, In_2Se_3 and In_4Se_3) and exhibit both lamellar (2D) and three-dimensional (3D) structures. In order to confirm the resulting compound stoichiometry after our growth procedure we carried out scanning electron microscopy (SEM) and energy-dispersive X-ray spectroscopy (EDS), revealing the morphology and atomic content of our sample. In Figs. S1(a-b), we present both low- and high-magnification SEM images. EDS analysis is shown in Fig. S1c.

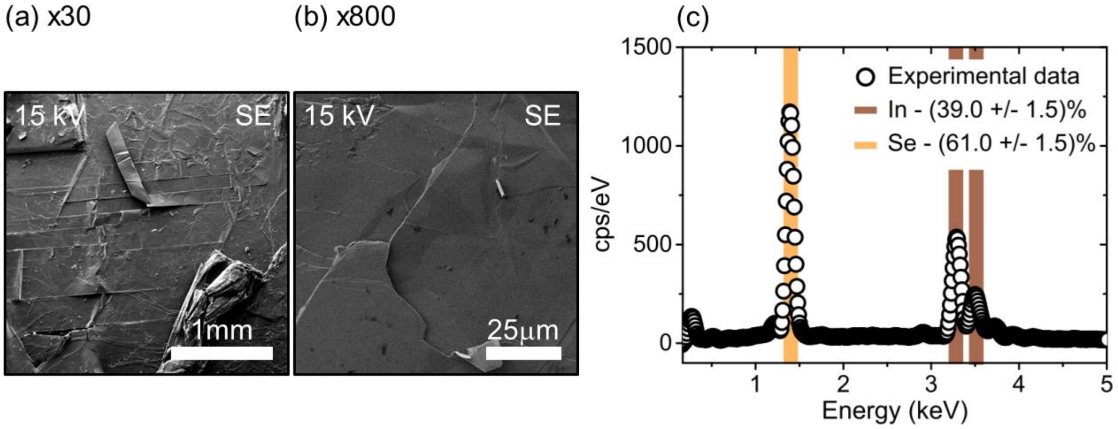

Figure S1: SEM images using secondary electrons (SE) detector at 15 kV **(a)** with x30 and **(b)** x800 magnification. Lamellar structures are observed at the surface (and similarly along the sample extension of few millimeters size). **(c)** Average of 15 EDS spectra obtained in distinct regions of our sample. The atomic ratio is approximately 40% In to 60% Se (2:3), as expected for In_2Se_3.

1.2 X-ray Photoelectron Spectroscopy Analysis

To obtain additional chemical and oxidation states for our In_2Se_3 sample, an initial XPS survey encompassing binding energies from zero to 755 eV was carried out.

This XPS survey is depicted in Fig. S2a, and the element peaks are highlighted in pink for oxygen, brown for indium, orange for selenium and grey for carbon. The binding energies for such elements are listed in Table S1. In Fig. S2(b-d), we show the indium, selenium and oxygen peaks and a theoretical fit for XPS data.

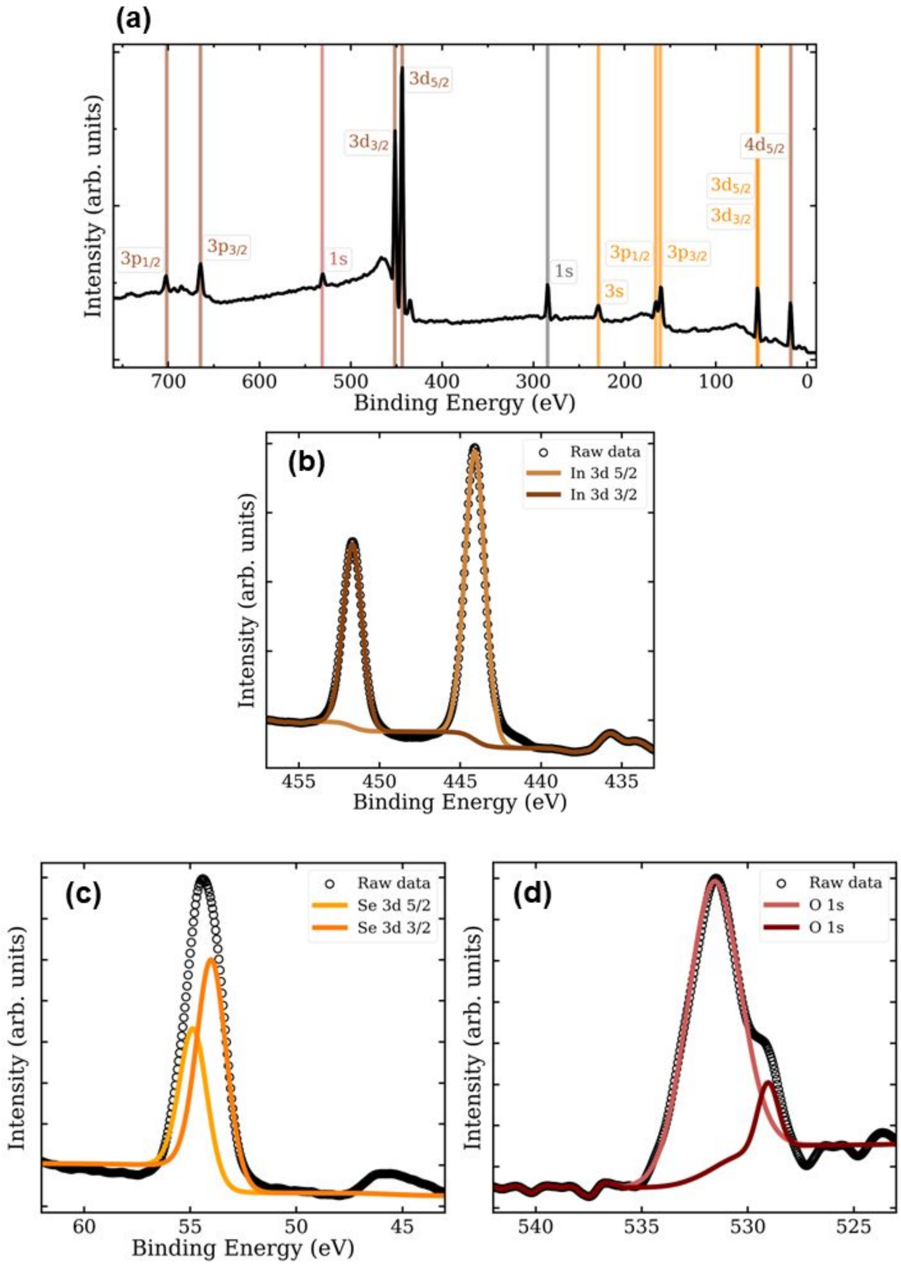

Figure S2. (a) XPS survey with peaks identified as a pink line for oxygen, brown lines for indium, orange for selenium and gray for carbon, which appears due to the conductive support double-side tape. **(b)** XPS data and theoretical fit of characteristic peaks for the elements indium (3d) and **(c)** selenium (3d). **(d)** two oxygen 1s peaks at

529.1 eV (from oxygen in In_2O_3) and 531.5 eV (from O_2). Those peaks may indicate a limited oxidation of the sample surface. This oxide phase has a low concentration since its intensity is reduced if compared to the other oxygen peak.

Element	Spectral Line	Bond	Binding Energy (eV)
C	1s		284.5
O	1s		531.5
	1s	In_2O_3	529.1
In	3s		827.3
	$3p_{1/2}$		702.2
	$3p_{3/2}$		665.4
	$3d_{3/2}$		451.7
	$3d_{5/2}$		444.1
	$4d_{5/2}$	In_2Se_3	18.0
Se	3s		230.1
	$3p_{1/2}$		166.7
	$3p_{3/2}$		160.6
	$3d_{3/2}$		54.0
	$3d_{5/2}$	In_2Se_3	54.9

Table S1. List of identified peaks from the XPS survey carried out in this work.

1.3 X-ray diffraction

Table S2 lists the volume proportion of each crystallographic phase retrieved from our Rietveld analysis. Structural parameters from XPD data fits are also provided for each phase.

Polymorph	*Structure*	*Space group*	*a (Å)*	*b (Å)*	*c (Å)*	*α(°)*	*β(°)*	*γ(°)*	*Proportion*
α-In_2Se_3	rhombohedral	R3m	9.86	9.82	9.96	24.43	23.68	23.59	27%
β-In_2Se_3	rhombohedral	R-3m	9.53	9.90	9.78	26.20	21.91	24.47	47%
δ-In_2Se_3	trigonal	P-3m1	4.00	3.99	9.61	84.75	95.30	122.17	20%
ε-InSe	trigonal	P-6m2	4.20	3.76	17.96	84.61	76.16	118.61	6%

Table S2. Crystallographic data obtained using the Rietveld method for powder diffraction data.

2.0 Electronic Characterization

2.1 Density Functional Theory Calculations

Electronic band structure and density of states calculations were performed using the Heyd-Scuseria-Ernzerhof (HSE06) hybrid functional within the density functional theory (DFT) framework. In Fig. S3(a-d), we present the electronic band structure and the respective electronic density of states calculations for α(3R), α(2H), β(3R)- and δ-In_2Se_3. All these layered In_2Se_3 structures exhibit a semiconductor band structure, with a fundamental indirect bandgap in bulk material. DFT calculations were not carried out for β(2H)-In_2Se_3 due to the lack of reference crystallographic information.

The α(3R)-In_2Se_3 structure shows the largest bandgap among the layered In_2Se_3 structures, approximately 1.20 eV, as shown in Fig S3a. The conduction band minimum (CBM) and the valance band maximum (VBM) are situated in the Γ point and Γ-L direction, respectively. Similarly, α(2H)-In_2Se_3 (Fig. S3b) exhibits a bandgap similar to that of the rhombohedral α polytype, accounting for 1.19 eV. The VBM is along the Γ-M direction and the CBM is at the Γ point. For β(3R)-In_2Se_3 (Fig. S3c), we observe a reduction in the bandgap value compared to the α-In_2Se_3 structures. We obtained a bandgap of 0.52 eV, where the CBM is located at the L point and the VBM is located between Γ and $S_0|S_2$ points. For the δ-In_2Se_3 (Fig. S3d), which is a trigonal stacking variant of β-In_2Se_3, the CBM occurs at the M point and the VBM occurs between K and Γ points, with a bandgap of 0.32 eV. For these α and β polytypes, one observes minor

electronic variations, which might not be directly observed in a non-local experimental setup.

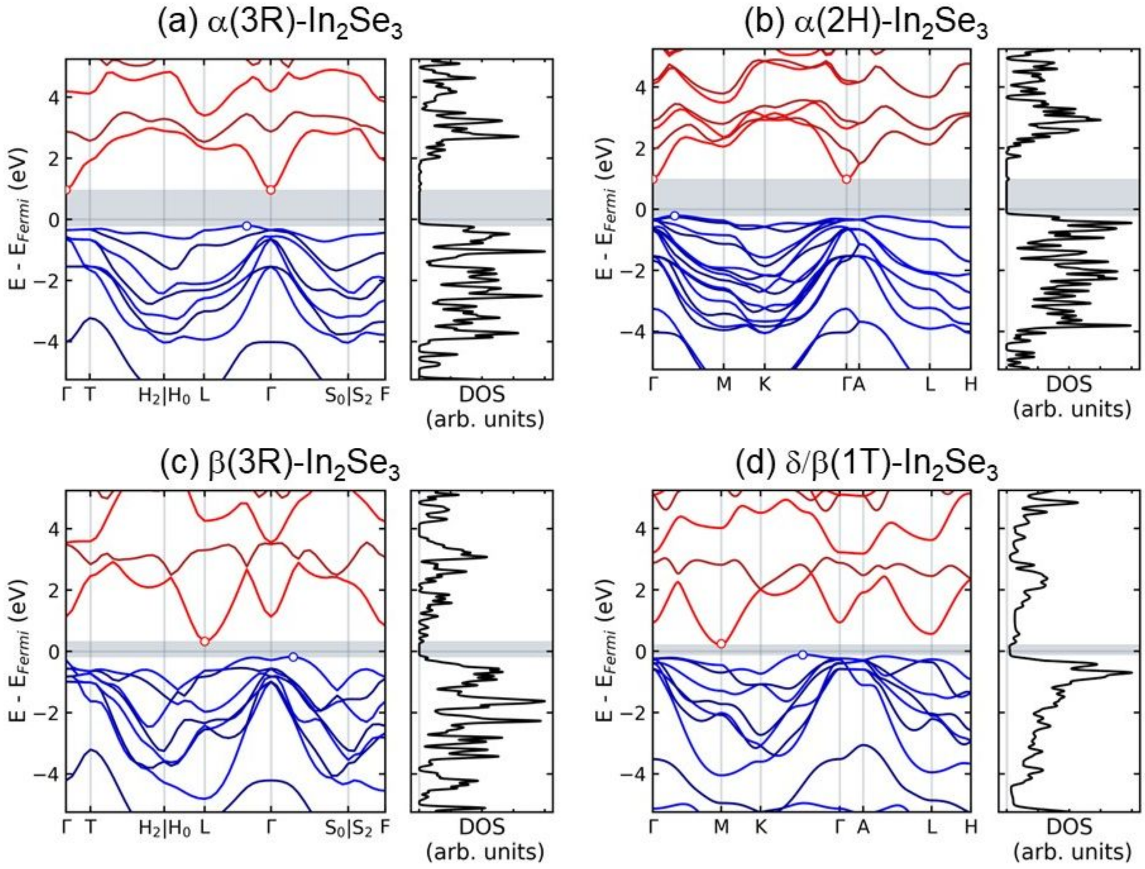

Figure S3. Electronic band structure and electronic density of states (right panel insets) calculations using DFT (HSE06 functional) for: **(a)** α(3R)-In_2Se_3, **(b)** α(2H)-In_2Se_3 **(c)** β(3R)-In_2Se_3 and **(d)** δ-In_2Se_3. These structures are semiconductors with indirect bandgap in their bulk form.

In Fig. S4(a-b), we present the electronic band structure and electronic density of states calculations for ε-InSe. In Fig. S4c, we present the tunneling spectrum for ε-InSe, which can be easily correlated to the calculations.

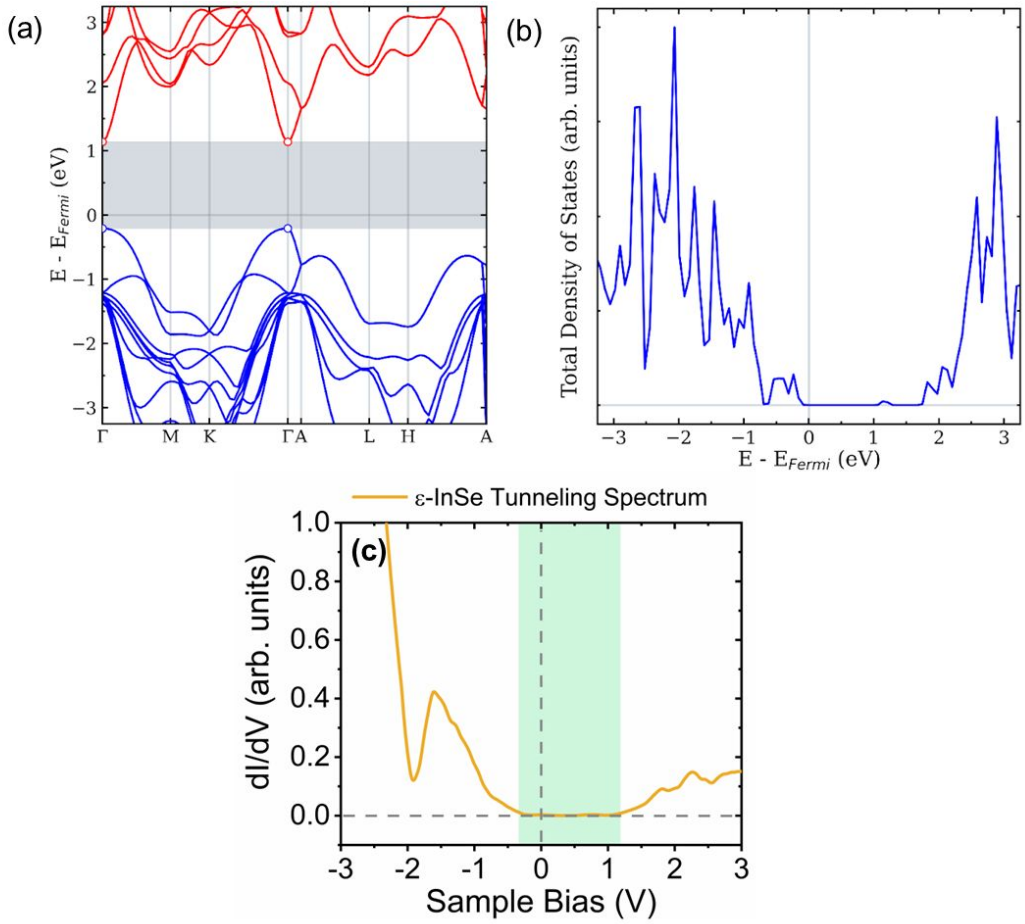

Figure S4. (a) Electronic band structure calculation for ε-InSe. The calculated electronic bandgap is 1.42 eV. **(b)** Electronic density of states calculations. **(c)** Tunneling spectrum associated with ε-InSe.

2.2 Angle-Resolved Photoemission Spectroscopy Analysis

The DFT calculations showed that the β- and δ-In_2Se_3 polymorphs have a very similar dispersion relation, differing only slightly in the shape of some bands as seen in Fig. S5(a, b, d, e). It is possible to notice that the ARPES measurement shown in Fig. S5(c, f) exhibits good agreement for both polymorphs but cannot provide a clear distinction between the abovementioned phases.

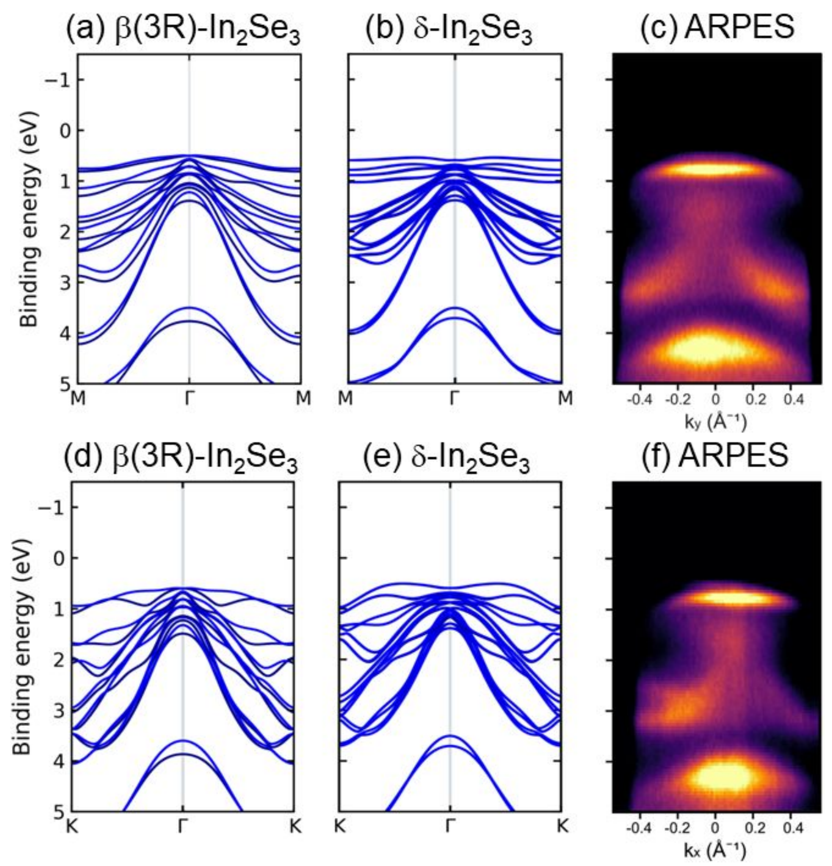

Figure S5. Comparison of the β(3R) and δ electronic band structure structures and ARPES measurements. **(a)** β(3R)-In_2Se_3 and **(b)** δ-In_2Se_3 at M-Γ-M. **(c)** ARPES measurements in k_y. **(d)** β(3R)-In_2Se_3 and **(e)** δ-In_2Se_3 at K-Γ-K. (d) ARPES measurements in k_x. One observes that measurements are not fully conclusive in phase determination, mostly due to the co-existence of sub-micrometer crystalline grains of β(3R) and δ-In_2Se_3 (comprising 67% of our crystal), along with similar band structure for both structures.

2.2 Scanning Tunneling Microscopy/Spectroscopy

Fig. S6 shows the direct STS data analysis, not considering the weighted XPD phase proportion (main manuscript). Since STM/STS are local and spatially restricted techniques, the relative presence of each phase does not match the bulk XPD analysis.

Nevertheless, the energy gaps extracted from STS using the direct (not-normalized) ensemble of measurements are compatible with the bulk-weighted data shown in the manuscript text.

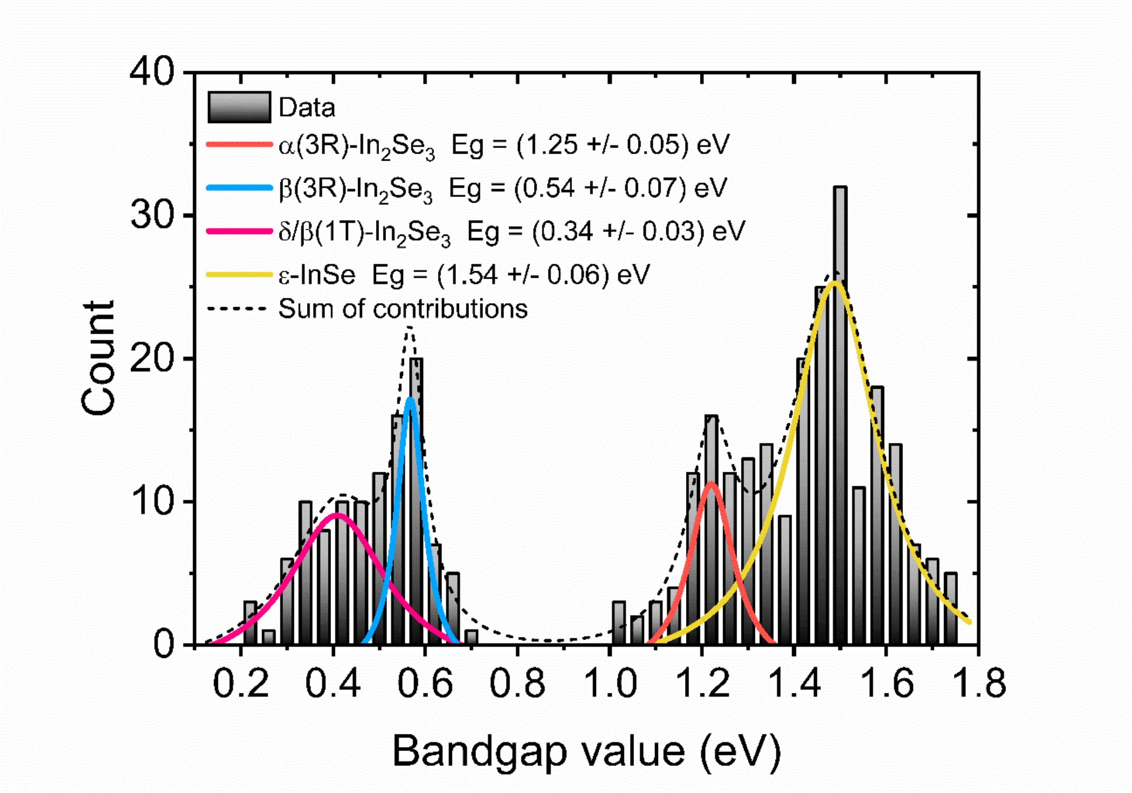

Figure S6. Bandgap values histogram obtained using scanning tunneling spectroscopy, not normalized from powder X-ray relative phase volume values. We acquired a substantial number of spectra from ε-InSe due to extensive spectra acquisition in a specific region (grain) of the sample.

We obtained surface termination information using STM and STS, as illustrated in Fig. S7. The height profile in Fig. S7a reveals regions where the height corresponds to the composition of In_2Se_3 and ε-InSe layers. Through STS, we confirmed that these ε-InSe layers are present on the surface of the image. The other steps are associated with α(3R)-In_2Se_3. We used green to represent the ε-InSe terminations and red for the α(3R)-In_2Se_3 terminations.

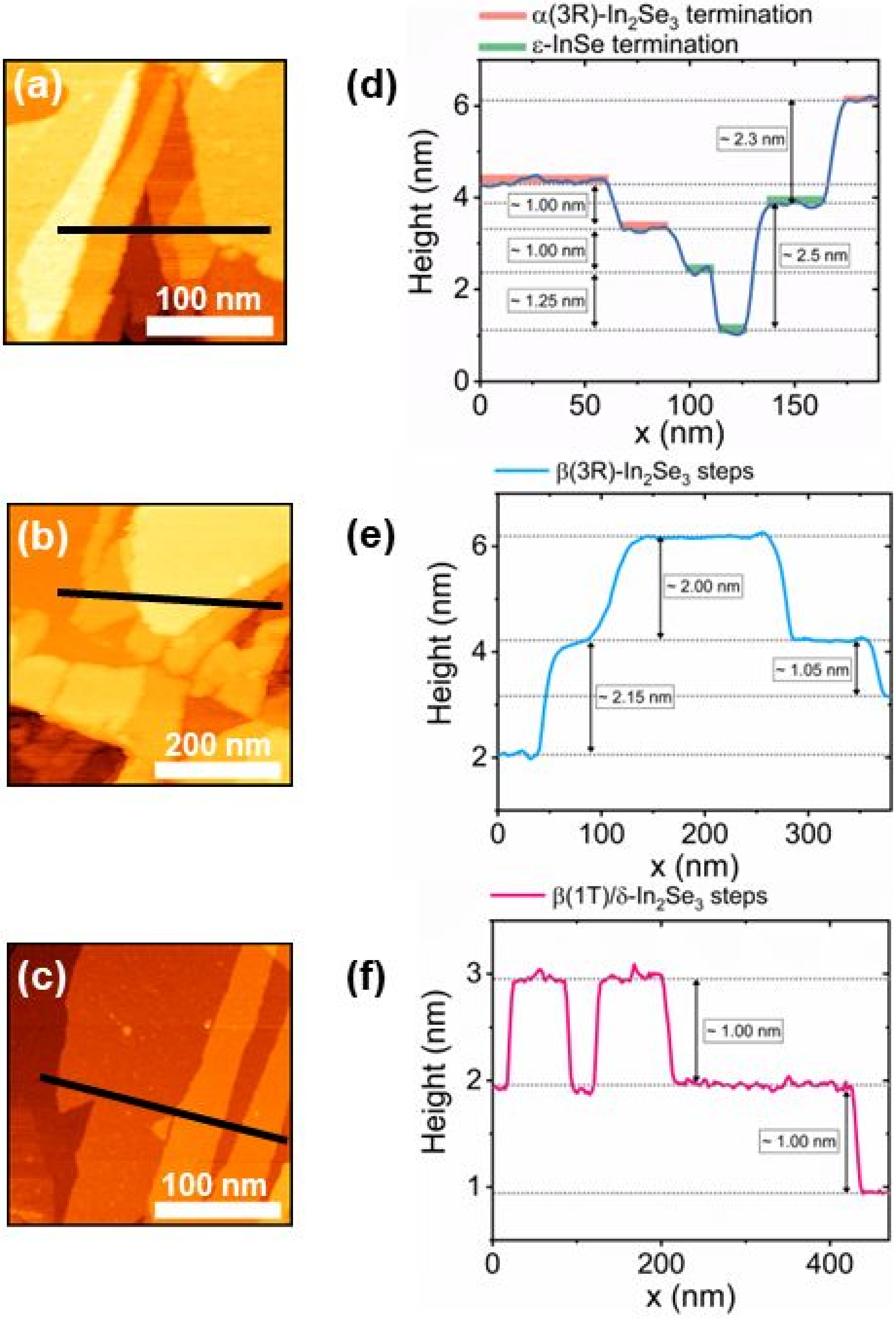

Figure S7. (a), **(b)**, and **(c)** show STM images with line profiles taken from right to left to measure the height of the steps. **(d)**, **(e)**, and **(f)** display the step height curves corresponding to images (a), (b), and (c) respectively. Surface termination information was obtained in conjunction with the analysis of the dI/dV curves for each region**.**

Additional atomic force microscopy measurements of ε-InSe region. We obtained a step height of approximately 0.61 nm.

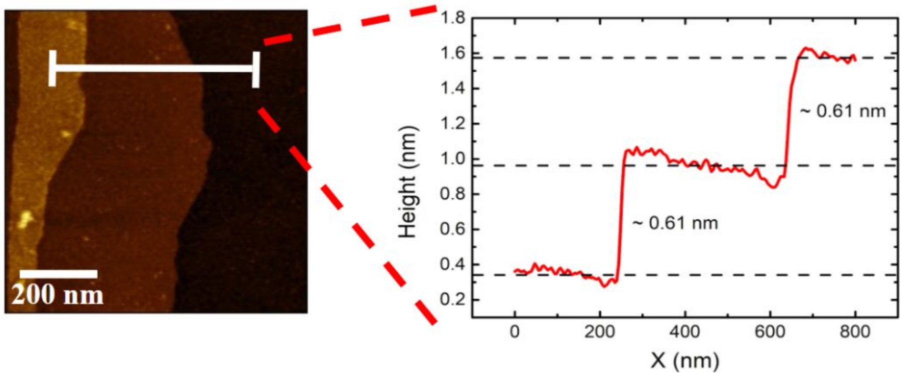

Figure S8. ε-InSe region probed using atomic force microscopy. Height measurements differ from expected values for In_2Se_3 structures but align correctly with the expected height value for the ε-InSe structure.
